# Cow’s milk allergen β-lactoglobulin immunoreactivity affected by pulsed light treatment

**DOI:** 10.1186/2045-7022-5-S3-P50

**Published:** 2015-03-30

**Authors:** Janire Orcajo, Iñigo Martinez de Marañon, Maria Lavilla

**Affiliations:** 1 Fundacion Azti-Tecnalia Fundazioa, Derio, Spain

## 

Cow’s milk allergy (CMA) is one of the most common food allergies especially in early childhood. One of the major allergens in cow’s milk is the beta-lactoglobulin (β-lg).

High temperature processes are known to denature whey proteins causing changes in their nutritional, organoleptic or technological properties. Consequently, an important challenge is to develop non-thermal technologies which can prevent adverse thermal effects and produce safe food products. One of these non-thermal technologies, Pulsed Light (PL), is a technology that consists of a successive repetition of short duration and high power flashes of broadband emission light (200-1000 nm) that has been shown to be effective in inactivating a wide broad of microorganisms.

Proteins are among the major targets for photo-induced modifications due to the abundance of endogenous chromophores within their structure. PL treatments were demonstrated to cause milk protein aggregation by disulfide bonds without further significant changes in protein components and induce conformational changes in β-lg. Despite these findings, the effect of PL on the allergenicity of proteins is still a matter of speculation.

Thus, the aim of this study is to determine whether such treatment affects the immunoreactivity of the β-lg. PL treatments of β-lg from bovine milk dissolved in several media at different pH were performed in a Xenon lamp with a fluence of 4, 8, 12 and 16 J cm^-2^. After treatments, samples were analyzed by native-PAGE and an enzyme-linked immunosorbent assay (ELISA) with polyclonal IgG, usually used in β -lg detection kits.

Results in native-PAGE showed a breakdown of the β-lg appearing some bands of lower molecular weight. The ELISA demonstrated a gradual decrease interaction allergen-IgG with the intensity of fluence applied.

As results suggest, a conformational change in the protein is provoked by PL technology covering up the detection of this allergen. It will be interesting to discover whether epitopes recognized by specific IgE antibodies (responsible of triggering type I hypersensitivity responses) in the sera of patients recognize the same epitopes as those recognised by the IgG used in this study.

